# Overlapping Tubulointerstitial Lupus Nephritis and Immunoglobulin G4-Related Disease: A Case Report

**DOI:** 10.7759/cureus.40664

**Published:** 2023-06-19

**Authors:** Ioannis Karageorgiou, Ashbina Pokharel, Indira Acharya, Ashbita Pokharel, Stavros Karageorgiou

**Affiliations:** 1 Internal Medicine, Beaumont Hospital, Royal Oak, USA; 2 Internal Medicine, MedStar Union Memorial Hospital, Baltimore, USA; 3 Pathology, Beaumont Hospital, Royal Oak, USA; 4 Rheumatology, University of Athens Medical School, Athens, GRC

**Keywords:** s: sle, igg4-rd, tubulointerstitial nephritis, igg4 disease, systemic lupus erythematosus

## Abstract

Immunoglobulin G4-related disease (IgG4-RD) in conjunction with systemic lupus erythematosus (SLE) is a rare occurrence. In this case report, we present the case of a 50-year-old female who was diagnosed with SLE based on clinical and laboratory criteria. The patient exhibited pericardial effusion necessitating pericardiocentesis, pleural effusion requiring thoracentesis, and impaired renal function necessitating dialysis. Renal biopsy revealed findings consistent with tubulointerstitial lupus nephritis and IgG4-related disease. Additionally, elevated levels of serum IgG4 were detected. The patient received intravenous pulse dose steroids and oral steroids, which were tapered gradually, followed by daily hydroxychloroquine treatment and two doses of rituximab every two weeks. Consequently, the patient experienced an improvement in renal function and no longer needed dialysis. To our knowledge, only a few reports of this overlap exist. This late diagnosis of SLE could be explained by the fact that IgG4 is associated with milder renal disease in lupus patients, due to its inability to activate the classical complement pathway. IgG4-RD/SLE overlap patients usually respond well to a combination of steroids and other immunosuppressants used to treat SLE. However, our experience with treating this disease overlap remains limited due to its extreme rarity.

## Introduction

Systemic lupus erythematosus (SLE) is an autoimmune disorder that frequently impacts American women of African descent who are in their reproductive years [1]. The diagnosis of SLE necessitates the fulfillment of clinical and serological criteria, including the presence of specific markers and autoantibodies [1]. A significant proportion of individuals with SLE, ranging from 30% to 50%, experience clinically observable kidney damage most commonly referred to as lupus nephritis (LN) [1]. LN is characterized by the deposition of immune complexes within the glomeruli, with IgG serving as a crucial constituent of these complexes [2]. Subsequently, IgG stimulates the activation of the complement system, leading to kidney injury.

Immunoglobulin G4-related disease (IgG4-RD) is a systemic fibroinflammatory ailment characterized by the infiltration of lymphoplasmacytic cells into various tissues, along with storiform fibrosis, obliterative phlebitis, and elevated levels of IgG4 in the bloodstream [3]. The diagnosis of IgG4-RD relies on clinical manifestations, biopsy findings, and immunological parameters [4]. Glucocorticoids represent the primary treatment modality for this condition, although additional immunosuppressive medications may be employed depending on the severity and presentation of the disease [4].

## Case presentation

**Figure 1:**
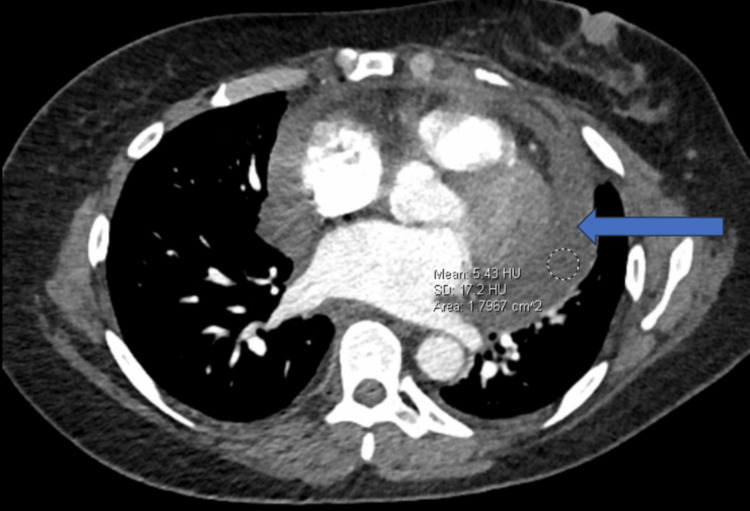
CT of the chest demonstrating pericardial effusion (blue arrow).

**Figure 2:**
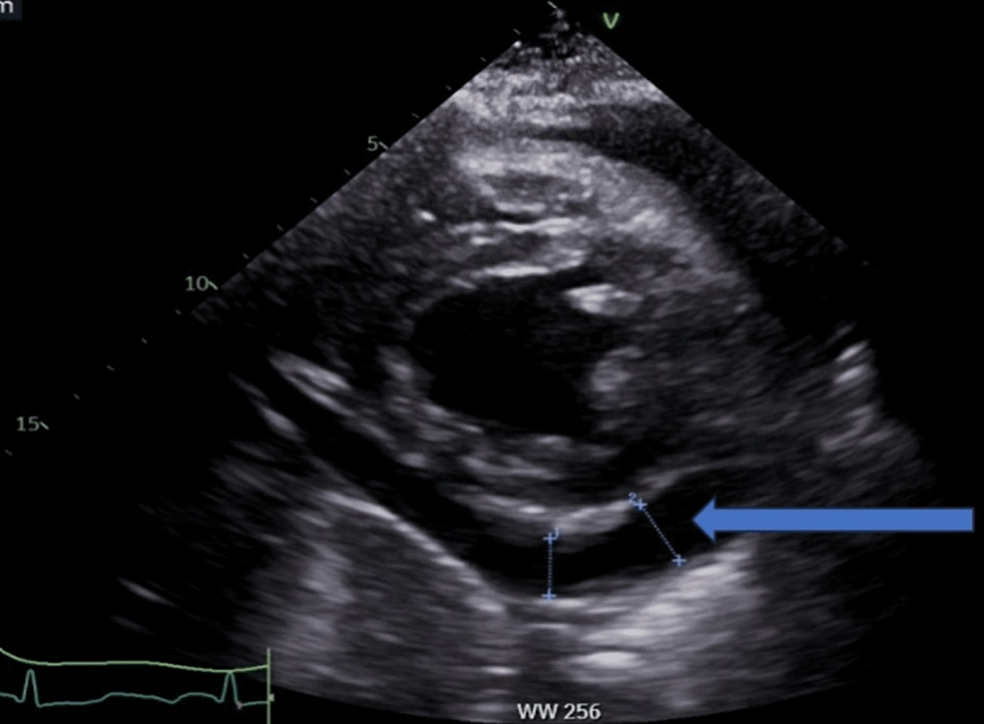
Echocardiogram demonstrating pericardial effusion (blue arrow).

**Figure 3:**
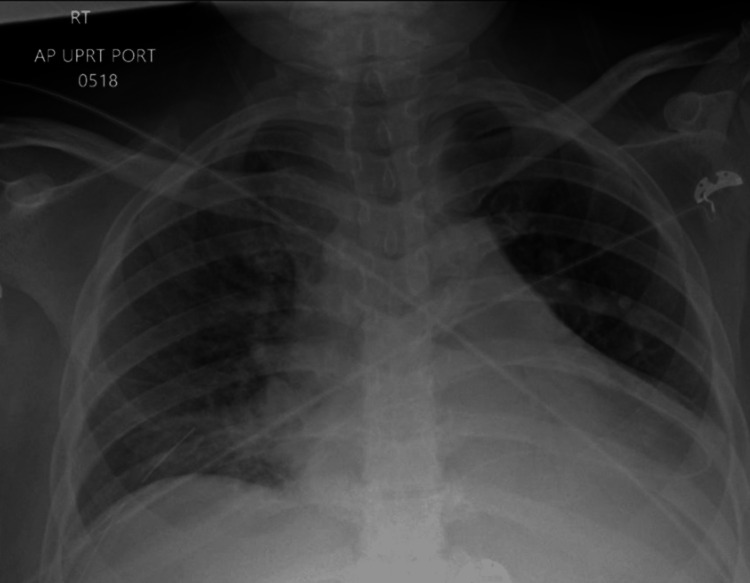
Chest X-ray showing fluid overload (worse on the left side).

A 50-year-old female patient was admitted to our medical facility to place a Quinton catheter and initiate dialysis treatment. The patient had a previous history of deep vein thrombosis in the right lower extremity and was on anticoagulation therapy. She initially presented to the hospital due to symptoms of shortness of breath and chest pain. A computed tomography (CT) chest angiogram revealed a significant pericardial effusion, as depicted in Figure 1. Subsequent echocardiography confirmed the presence of pericardial effusion with tamponade physiology, as shown in Figure 2. A pericardial window procedure was performed to address this condition. A chest X-ray demonstrated bilateral pleural effusions, as illustrated in Figure 3, treated with a thoracentesis. Despite these interventions, the patient continued to experience fluid overload and was started on diuretics. Unfortunately, her kidney function deteriorated further upon the administration of diuretics. The patient's creatinine level rose to 5.21 mg/dL (reference range: 0.5-1.1 mg/dL), with a baseline creatinine of 1.4 mg/dL three months prior. The estimated glomerular filtration rate (eGFR) dropped to 9 mL/minute/1.73 m^2^, compared to a baseline eGFR of 50 mL/minute/1.73 m^2^ three months earlier. Blood urea nitrogen (BUN) levels also increased to 57 mg/dL (reference range: 7-25 mg/dL), in contrast to 32 mg/dL three months prior. Consequently, the patient was transferred to our center for dialysis treatment.

**Figure 4:**
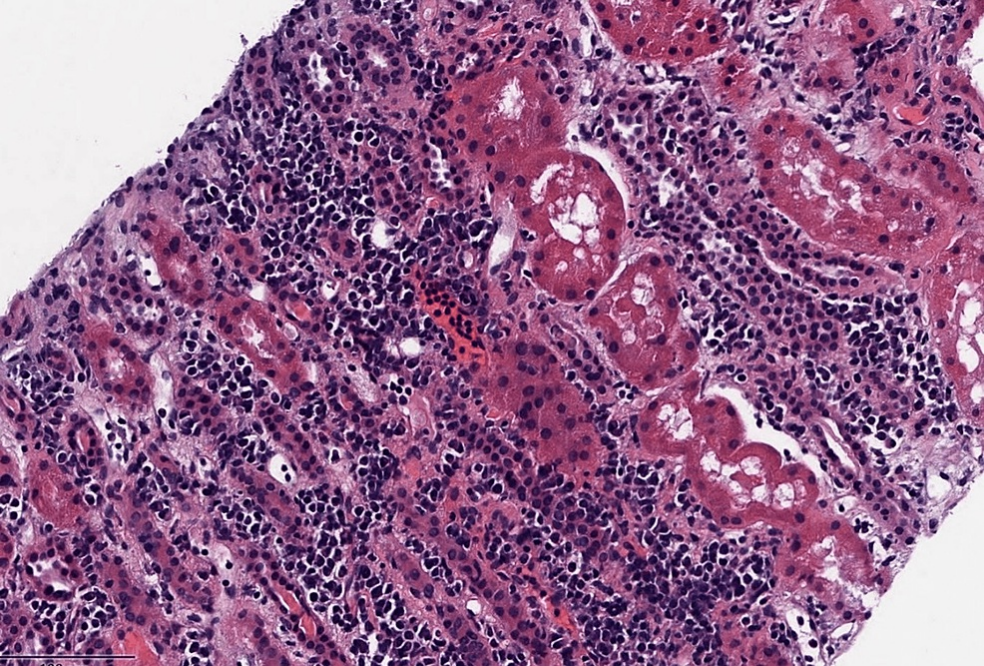
Renal biopsy findings. Light microscopy (hematoxylin and eosin staining) demonstrates lymphoplasmacytic interstitial infiltrate.

**Figure 5:**
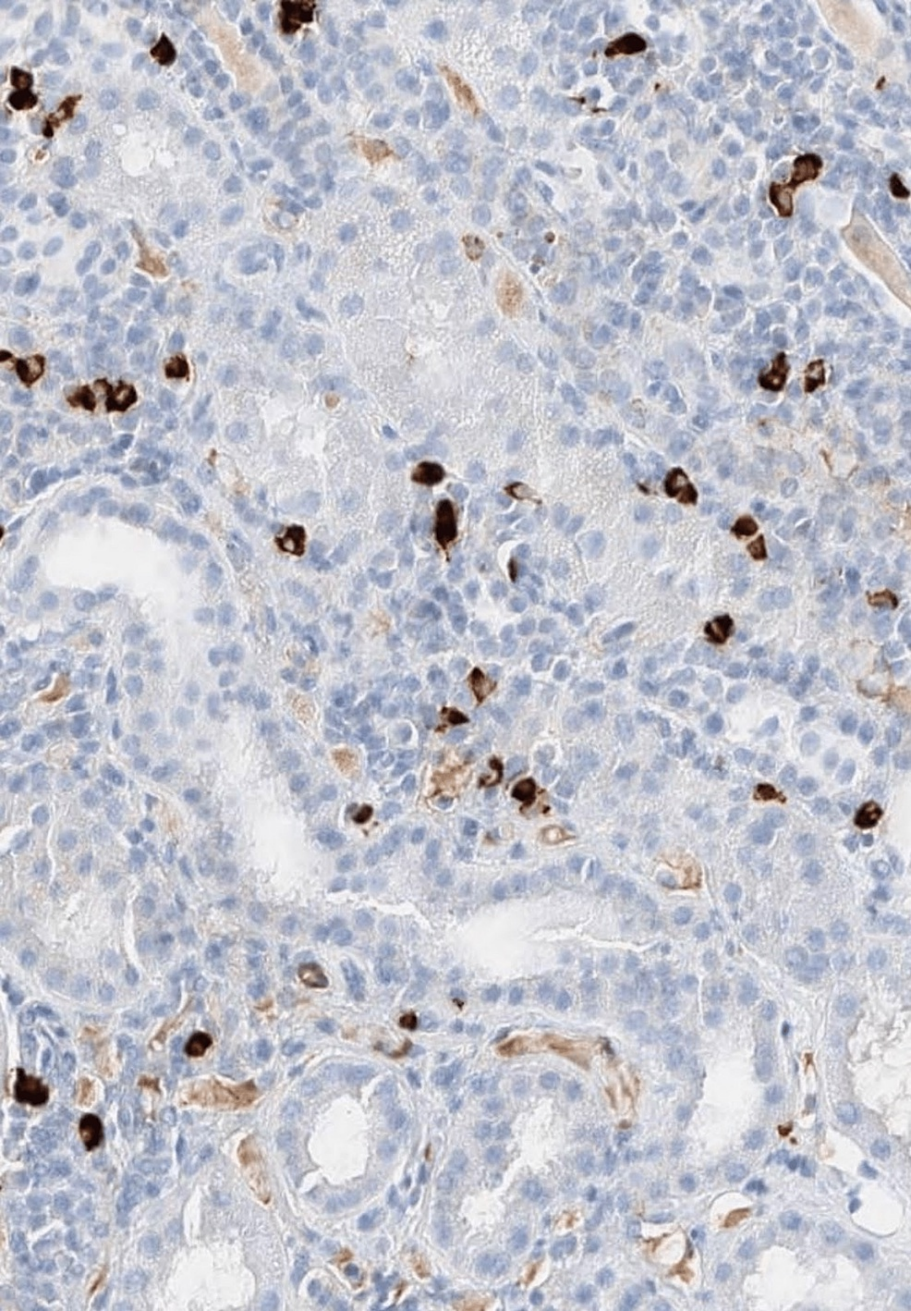
Renal biopsy findings. IgG4 immunostain shows an increased number of IgG4-positive plasma cells. IgG4: Immunoglobulin G4.

Upon arrival at our facility, the patient's vital signs were as follows: blood pressure of 127/68 mmHg, heart rate of 85 beats per minute, respiratory rate of 18 breaths per minute, and oxygen saturation of 100% on 2 liters of supplemental oxygen. Physical examination revealed crackles upon lung auscultation and bilateral lower extremity pitting edema graded as 2+. Lab investigations demonstrated a white cell count of 3.9 billion/L (reference: 3.3-10.7 billion/L), a hemoglobin level of 8.5 g/dL (reference: 12.1-15 g/dL), and a platelet count of 302 billion/L (reference: 150-400 billion/L). Urinalysis exhibited the presence of more than 20 hyaline casts per low-power field (reference: negative per low-power field) and a protein level of 100 mg/dL (reference: negative). Other immunological laboratory results are shown in Table 1. The findings from the chest X-ray were consistent with bilateral pleural effusions. The patient underwent dialysis, resulting in improved volume status and renal function. Surprisingly, a left kidney biopsy revealed findings of tubulointerstitial nephritis with features suggestive of IgG4-related disease, as depicted in Figure 4. Immunofluorescence microscopy indicated lupus glomerulonephritis WHO class II, as shown in Figure 5. 

**Table 1:** Immunological workup on presentation IFA: indirect fluorescent antibody

Variable	Result	Reference Range
Antinuclear antibody	≥ 1:1280	< 1:160
Anti-double-stranded DNA, IFA	1:320	< 1:10
Complement C3	35 mg/dL	82-193 mg/dL
Complement C4	10 mg/dL	10- 43 mg/dL
Anti-nuclear cytoplasmic antibody	< 1:20	< 1:20
Immunoglobulin G (IgG)	3,227 mg/dL	550-1650 mg/dL
Immunoglobulin G1	1,886 mg/dL	341-894 mg/dL
Immunoglobulin G2	362 mg/dL	171-632 mg/dL
Immunoglobulin G3	136 mg/dL	18-106 mg/dL
Immunoglobulin G4	228 mg/dL	2.4-121 mg/dL
Sjogren’s syndrome A Antibody, IgG	395 AU/mL	< 100 AU/mL

Additionally, the patient's lab work demonstrated elevated IgG4 levels, as outlined in Table 1, suggesting the presence of IgG4-related disease. Treatment was initiated with Plaquenil at 200 mg twice daily, accompanied by a pulse of intravenous corticosteroids administered at 1 g daily for three days. Following the completion of intravenous steroid therapy, the patient was transitioned to oral prednisone at a dose of 30 mg twice daily, with plans to taper the dosage by 10 mg per week. After discharge, the patient received rituximab at 1 g every two weeks. During a follow-up visit one month later, the patient was no longer dependent on dialysis and was maintained on a daily dose of 30 mg of steroids.

## Discussion

SLE is a chronic immune-mediated disorder with an unknown etiology that affects multiple bodily systems. Virtually all organs can be affected by this condition [1]. LN significantly contributes to the mortality of SLE patients. Renal complications occur in 30-50% of SLE patients, and approximately 30% of individuals develop end-stage renal disease within 10-15 years after diagnosis, necessitating kidney replacement therapy [1].

IgG4-RD is an immune-mediated condition characterized by the formation of masses that cause irreversible organ damage and even mortality if left untreated [4]. This disorder is characterized by numerous IgG4-positive plasma cells in affected tissues and the development of fibrosis. The initial description of this disorder dates back to 2003, and since then, its recognition has been increasing globally [4]. Various aspects related to the pathophysiology, accurate diagnosis, and optimal management of this disease are areas of ongoing research [4].

This case report presents a rare occurrence of concurrent SLE and IgG4-RD. As far as we know, only a limited number of case reports documenting the coexistence of these two conditions have been published [3,5-7]. Our case involved a 50-year-old woman who required dialysis initiation and was subsequently diagnosed with SLE and IgG4-related tubulointerstitial nephritis. Tan et al. reported a case of tubulointerstitial nephritis with a storiform pattern and infiltration of IgG3-positive plasma cells in a 67-year-old man [5]. Takanashi et al. described an overlap in a 46-year-old man with Klinefelter syndrome, presenting with obliterative phlebitis and progressive decline in renal function [6]. Naramala et al. presented the case of a 63-year-old woman with eosinophilia and diffuse lymphadenopathy [3]. Yamamoto et al. presented a 58-year-old woman with a photosensitive rash and lymphadenopathy in the cervical, mediastinal, and axillary regions [7]. It is worth noting that the average age of these five cases, 56.8 years, is significantly higher than the typical age of onset for SLE, which predominantly affects young African American females of reproductive age [1]. This age range aligns more closely with the demographic profile of patients with IgG4-RD, which primarily affects middle-aged and older individuals [4]. Renal involvement appears to be a common feature in all the reported cases, evident through microscopic examination or imaging studies [3,5-7]. Other manifestations of the disease overlap include skin involvement and lymphadenopathy, which are well-documented features of IgG4-RD [6,7]. In our case, renal involvement appeared to be the sole manifestation of IgG4-RD and SLE.

Regarding renal involvement, sometimes it can be challenging to differentiate between IgG4-related tubulointerstitial nephritis and SLE-related tubulointerstitial nephritis because elevated IgG4 subclasses exist in classical SLE cases without IgG4-RD [2,8-15]. Interestingly, SLE cases demonstrating a predominance of IgG4 subclasses in renal biopsy specimens have shown a more favorable prognosis when compared to typical SLE patients [2,8,9,11-14]. This outcome can be attributed, at least in part, to the distinctive characteristics of the IgG4 molecule, including its inability to activate the classical complement pathway and its unique "fab-arm exchange" property, which inhibits the formation of large immune complexes [8,10,11].

Based on the above information, it is plausible that patients experiencing an overlap of IgG4-RD and SLE may exhibit a less severe disease course and potentially present later than the average SLE patient. The milder disease presentation could explain the elevated mean age observed in the reported cases. However, this hypothesis remains unverified and requires validation through larger-scale studies.

Regarding treatment, a combination of steroids and steroid-sparing agents was employed in most patients, which focused on managing the SLE component [3,5-7]. In our case, the patient was started on pulse-dose steroids and Plaquenil, followed by low-dose steroid maintenance and rituximab therapy. Other authors utilized prednisone, Plaquenil, cyclophosphamide, and mycophenolate mofetil (MMF) for induction and maintenance treatment (3,5,6). Yamamoto et al. reported the successful use of belimumab after the failure of prednisone and MMF therapy [7]. Therefore, tailoring the treatment approach based on the severity of the SLE component appears reasonable, and second or third-line agents commonly employed in SLE management may also be beneficial in these cases. While one of the cases mentioned a gradual steroid taper over five months with positive outcomes, the use of steroid tapering was not addressed in the other cases [3].

## Conclusions

SLE and IgG4-RD are immune system disorders with distinct clinical presentations, disease courses, epidemiology, and management approaches. Although rare, there have been documented cases of patients being affected by both conditions. While renal involvement appears to be the most prevalent manifestation, the skin, lymph nodes, and other organs can also be affected. Notably, older individuals are more frequently impacted by this overlap, potentially due to the interplay between the two diseases resulting in a milder presentation of SLE, consequently delaying diagnosis. This phenomenon could be attributed to the unique characteristics of IgG4, including its inability to activate the classical complement pathway and its capacity to inhibit the formation of large immune complexes. When encountering patients with SLE/IgG4-RD overlap, a treatment approach similar to that of SLE patients is typically employed, with a favorable outcome.
